# Extensor Tendon Transfers for Radial Nerve Palsy Secondary to Humeral Shaft Fracture

**Published:** 2018-09-21

**Authors:** Elizabeth A. Lucich, Matthew P. Fahrenkopf, John P. Kelpin, Teresa C. Hall, Viet H. Do

**Affiliations:** ^a^Spectrum Health/Michigan State University Plastic and Reconstructive Surgery Residency, Grand Rapids; ^b^Metro Health/University of Michigan Health Orthopaedic Residency, Wyoming; ^c^Orthopaedic Associates of Michigan, Grand Rapids

**Keywords:** extensor tendon transfer, radial nerve palsy, radial nerve laceration, humeral shaft fracture, rehabilitation following tendon transfer

## DESCRIPTION

A 62-year-old woman suffered a mid-humeral shaft fracture after a motor vehicle collision. She had a prolonged course in the intensive care unit, delaying the diagnosis of radial nerve palsy. Conservative management failed to demonstrate any regeneration of the nerve. Tendon transfers were planned and performed to restore thumb, wrist, and finger extension.

## QUESTIONS

1. How common is a radial nerve palsy following humeral shaft fracture?
2. What is the optimal timing for radial nerve repair following humeral shaft fracture?
3. What are the most common tendon transfers for radial nerve palsy?
4. What is the postoperative therapy after tendon transfers?

## DISCUSSION

Humeral shaft fractures have a high incidence of concomitant radial nerve palsy, given the intimate anatomic relationship between the two. The incidence of radial nerve injury following humeral shaft fracture is between 2% and 17%.^1^ More specifically, fractures of the middle and middle-distal part of the humeral shaft have a higher association with radial nerve palsy.^2^ The type of fracture can play into the likelihood of radial nerve palsy, as transverse and spiral fractures are more highly associated with radial nerve palsy than oblique or comminuted fractures.^2^

After a detailed sensory and motor examination, radial nerve palsy can be identified. The treatment of palsy can vary on the basis of the type of fracture. The standard of care for open humeral fractures with radial nerve palsy is immediate exploration and repair if found. However, with closed humeral fractures with radial nerve palsy, some advocate for a watchful waiting approach whereas others recommend immediate exploration. The spontaneous recovery rate for radial nerve palsy in closed humeral shaft fractures can be as high as 88%.^2^ In patients with closed fractures, the time to wait before surgical exploration is between 3 and 6 months, but more specific time frames can be estimated on a case-by-case basis, assuming nerves regenerate at 1 mm per day and then adding 30.^3^ If after 3 months, there is no recovery of function, some recommend proceeding with nerve transfer in which small muscular branches of the median nerve are transferred to the radial nerve.^4^ Nerve transfers can produce excellent results if performed within 10 months of the original injury.

**Figure 1:**
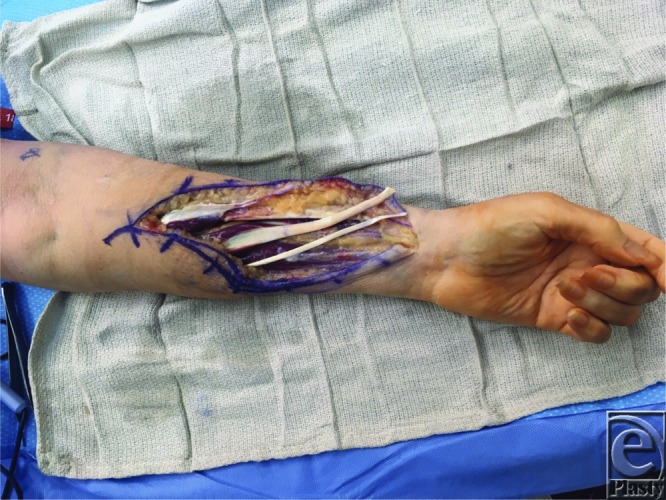
Volar forearm incision demonstrating harvest of the pronator teres, flexor carpi radialis, and palmaris longus tendons (top to bottom). Note the strip of periosteum included on the pronator teres tendon.

**Figure 2:**
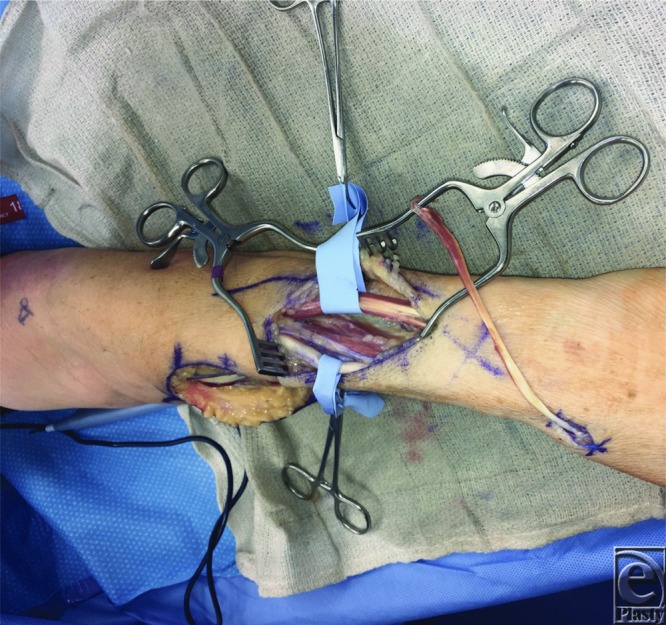
Dorsal forearm incision demonstrating identification and isolation of recipient tendons. Extensor digitorum communis (top), extensor carpi radialis brevis (bottom), and extensor pollicis longus (right).

**Figure 3:**
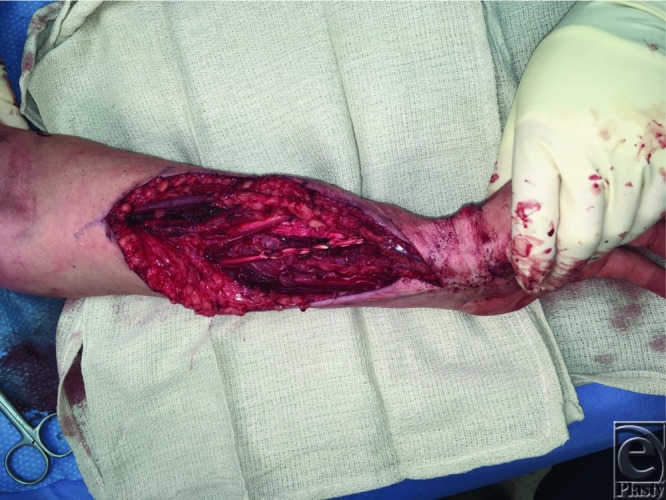
End-to-end tenorrhaphy of palmaris longus to extensor pollicis longus.

**Figure 4:**
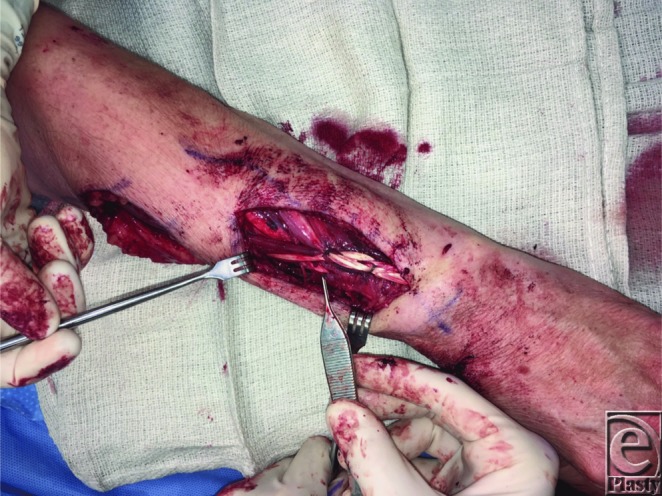
End-to-end tenorrhaphies of flexor carpi radialis to extensor digitorum communis (top) and pronator teres to extensor carpi radialis brevis (forceps).

However, if a radial nerve repair is not performed prior to 10 months, the motor end plate begins to degenerate, making any nerve repair obsolete. In these cases, the only option for a functional recovery is tendon transfer (Figs 1 and 2). Important considerations for successful tendon transfer are to ensure there are supple joints before surgery, availability of donors of adequate strength and excursion, creating a straight line of pull, and addressing only a single function per transfer.^5^ To restore wrist extension, the pronator teres is transferred to the extensor carpi radialis brevis (ECRB) tendon. If some radial nerve recovery is expected, this is performed in an end-to-side fashion so that once recovery is achieved, ECRB can resume wrist extension. However, if no radial nerve recovery is expected, the ECRB tendon is cut and the pronator teres is sutured in an end-to-end fashion. Thumb extension is restored using the palmaris longus tendon, if the patient has one, or the ring finger flexor digitorum superficialis (FDS) to the extensor pollicis longus (Fig 3). Finger metacarpophalangeal joint extension is most often restored in one of 3 ways: by using the flexor carpi radialis (FCR), flexor carpi ulnaris (FCU), or FDS to the extensor digitorum communis (EDC). The FCU transfer does have a negative drawback of allowing for radial deviation of the wrist, as the FCU is the only remaining ulnar-sided functional muscle after radial nerve palsy. Brand^6^ popularized the use of the FCR transfer to EDC (Fig 4), and Boyes and colleagues^7^ popularized the FDS transfer to EDC.

Regardless of the type of tendon transfer performed, postoperative therapy is the same. At first, an above-elbow splint should be placed with the elbow flexed, forearm pronated, and wrist extended. After 4 weeks, mobilization begins but should keep tension off all the transferred tendons. Muscle activation begins at week 6, but again tension should be kept to a minimum on the transferred tendons and synergistic action is discouraged. Strengthening exercises should begin at week 8, with full activity resumed at week 12.^8^

Radial nerve palsy can be a devastating injury. Tendon transfers allow for patients to regain some functional use of the hand and are an excellent option for patients. Tendon transfers require a precise knowledge of anatomy and a multidisciplinary team with occupational therapists postoperatively to ensure the best results.
